# A robotic and high-throughput X-ray micro-computed tomography workflow

**DOI:** 10.1107/S1600577526005539

**Published:** 2026-06-22

**Authors:** Xiaoyang Liu, Alex Lavens, James Bennett O’Sullivan, Alan Kastengren, Andrew T. Townsend, Jason G. Toyoda, Hasitha Wijesuriya, Tamas Varga, Karolina Michalska

**Affiliations:** a Advanced Photon Source Argonne National Laboratory Lemont IL60439 USA; b Earth and Biological Sciences Directorate, Pacific Northwest National Laboratory, Richland, WA, USA; Brazilian Synchrotron Light Laboratory, Brazil

**Keywords:** X-ray imaging, microtomography, high-contrast samples, automation, high-energy studies

## Abstract

With the recent upgrades of synchrotron facilities and an increasing demand for artificial intelligence (AI)-ready scientific datasets, there is a growing interest in high-throughput and automated experimental workflows to accelerate large-scale data-driven research. In this work, we demonstrate a fully automated data collection workflow for synchrotron micro-computed tomography experiment on soil cores on beamline 7-BM at the Advanced Photon Source (APS), operating without expert supervision. The automation integrates sample exchange, multi-position movement and data acquisition. A self-contained robotic arm system is designed for rapid and reliable sample exchange. It accommodates various sample types and can be readily deployed across multiple APS beamlines. Additionally, the beamline has recently upgraded the sample stage stacks to enable a large travel range and an efficient macroscope-based imaging system, enabling characterization of highly attenuating and large samples. This work establishes a foundation for future closed-loop, adaptive and intelligent synchrotron micro-computed tomography experiments.

## Abstract 2

A high-throughput and automated experimental workflow for synchrotron X-ray micro-computed tomography is demonstrated. The system integrates automated robotic sample exchange and data acquisition, enabling large-scale and data-driven research.

## 1. Introduction

X-ray computed tomography (CT), as a non-destructive imaging technique, enables direct visualization and quantitative analysis of the three-dimensional (3D) and interior morphology of an object (Stock, 2020 ▸; Maire & Withers, 2014 ▸; Withers *et al.*, 2021 ▸), making it particularly valuable for the study of complex biological systems (Miyashita *et al.*, 2025 ▸; Schmeltz *et al.*, 2024 ▸; Deleeuw *et al.*, 2023 ▸) and plants (Østerlund *et al.*, 2025 ▸; Duddek *et al.*, 2024 ▸), and in earth sciences (Pincus *et al.*, 2023 ▸; Rippner *et al.*, 2022 ▸; Lee *et al.*, 2025 ▸) and materials science (Huang *et al.*, 2025 ▸; Yoon *et al.*, 2025 ▸; Satapathy *et al.*, 2024 ▸; Peng *et al.*, 2025 ▸). As implemented at several synchrotron facilities worldwide, X-ray micro-CT provides spatial resolutions of 1 to a few micrometres (µm) with a field of view (FOV) ranging from 1 to a few millimetres (mm), and X-ray nano-CT using transmission X-ray microscopes (TXM) typically provides a spatial resolution of 30–60 nm with a FOV of 40–70 µm, with specific optomechanical breakthroughs leading to sub-10 nm resolution (De Andrade *et al.*, 2021 ▸; Zhang *et al.*, 2022 ▸; Chen *et al.*, 2023 ▸; Lin *et al.*, 2024 ▸; Mittone *et al.*, 2024 ▸). The high flux of synchrotron beamlines reduces the acquisition time compared with laboratory-based sources, which enables *in situ* and high-throughput studies (Ge *et al.*, 2018 ▸; Liu *et al.*, 2021 ▸). The upgrade to fourth-generation storage-ring sources provides a much greater scope for phase-based imaging techniques with the improved X-ray source size and coherence (Shi *et al.*, 2025 ▸).

With the recent source and instrumentation advancements, the number of samples that can be measured during a period of beam time is expected to increase substantially, facilitating data-driven materials discovery and quantitative studies of heterogeneous systems. A typical workflow on an X-ray micro-CT beamline requires users to mount and exchange every sample by physically going into the experimental hutch. If the sample is larger than the FOV, the users find the region of interest (ROI), set the scan parameters and start the data acquisition. If the sample is smaller than the FOV, the users align the sample ROI at the center of rotation (CoR) before the scan. The above steps need to be repeated for each sample, which is time-consuming and inefficient, so this manual workflow lags behind the experimental throughput supported by the upgraded storage ring. Thus, it is necessary to develop automation of sample exchange, sample alignment and data collection for effective utilization of beam time, particularly for studies requiring large numbers of samples for statistical analyses or artificial intelligence (AI) training datasets.

In this work, we have developed an automated and high-throughput X-ray micro-CT sample exchange and data-collection workflow on the 7-BM beamline at the Advanced Photon Source (APS). The high flux of the white beam, together with the integration of a high numerical aperture (NA) macroscope and flexible sample motor stacks, enables a large vertical travel range for multi-position acquisitions, *i.e.* sequential imaging of multiple ROIs via sample vertical translation. The experimental end station on 7-BM has been optimized to support efficient measurements of large or highly attenuating samples. These enhancements make the beamline well suited for large-scale and rapid tomographic image collection. To address the limitations of manual sample exchange in micro-CT measurements, we have incorporated the Tomography Object Mounter (TOM), a portable robotic arm system, into the workflow. The versatile and portable TOM system is designed to house all required hardware and control components, enabling straightforward deployment and integration across different beamlines. This approach not only eliminates the inefficiencies of manual sample handling, but also enhances the flexibility and scalability of automated experimental workflows, making it well suited for high-throughput synchrotron-based imaging applications.

We demonstrate the application of this end-to-end automation, including sample exchange, sample movement and tomographic data collection, by measuring a set of soil cores (diameter 76.2 mm) on 7-BM without human intervention. A key scientific motivation for this approach is to provide a high-throughput and high-resolution imaging capability to image multiple virtual ‘mini soil cores’ within a single large soil core, eliminating the need for performing destructive mini soil core sampling, which introduces sampling artifacts and bias. Overall, the developed workflow aims for a wide range of applications for attenuating and heterogeneous samples such as soil cores, dense geological materials and engineering components. The integrated infrastructure and operational throughput provide a practical foundation for future closed-loop and AI-guided synchrotron micro-CT experiments.

## 2. Method

### 2.1. Beamline setup and upgrades

The 7-BM beamline has undergone equipment updates associated with the facility upgrade. The micro-CT instrument on 7-BM uses a white beam which passes through beryllium windows (750 µm total thickness) and various filters (Cu, Ge and Si) with different thicknesses to filter out low-energy X-rays. The detector table and stage (Aerotech, Pittsburgh, USA) hold the X-ray imaging system which offers freedom of translation along the X-ray beam direction (*Z*). The sample-to-detector distance is adjustable from 80 to 960 mm, providing both absorption and in-line propagation phase contrast imaging modes. To accommodate large samples, the 7-BM sample motion stack includes a vertical translation stage with 230 mm of travel and a hexapod positioner (Aerotech, Pittsburgh, USA) with 50 mm of vertical travel. The air-bearing rotary stage is placed on top of the hexapod positioner. Two linear stages are mounted on top of the rotary stage to center the sample on the rotation axis. The hexapod positioner is used to move sample into or out of the FOV for flat-field acquisition.

There are two complementary imaging systems available on 7-BM. The first is a high-magnification microscope (Optique Peter, Lentilly, France) with an interchangeable objective lens, providing resolution down to ∼1 µm. The second is a high-efficiency X-ray imaging macroscope (Douissard *et al.*, 2012 ▸; Gasilov *et al.*, 2024 ▸) (Optique Peter, Lentilly, France) based on an infinity-corrected tandem optical design. The macroscope uses interchangeable objective pairs on the scintillator and detector sides, with the magnification defined by the ratio of their focal lengths (F), which gives discrete magnifications ranging from 0.5× to 2×, defined by the focal-length ratios of the coupled lens. The F = 200 mm and F = 100 mm objectives are used in this work, providing 2× magnification with an NA of 0.27. A 100 µm thick and 21 mm diameter LuAG:Ce scintillator is used in the system. Compared with a conventional 2× objective used in the high-magnification microscope (NA = 0.055), the newly implemented system provides a 25-fold higher light-collection efficiency, which is particularly beneficial for thick or highly attenuating samples as it significantly reduces data collection time with no deterioration of the signal-to-noise ratio. An Oryx ORX-10G-310S9M monochrome camera (FLIR, Wilsonville, USA) with a 31 MP Sony IMX342 complementary metal-oxide-semiconductor (CMOS) sensor (6464 × 4852 pixels, 3.45 µm pixel size) is used for both imaging systems. Overall, the enhanced capabilities on 7-BM support rapid and flexible micro-CT measurement, and provide a robust platform for large-scale imaging studies.

### 2.2. Tomography Object Mounter (TOM): portable robotic arm design and integration

**Figure 1:**
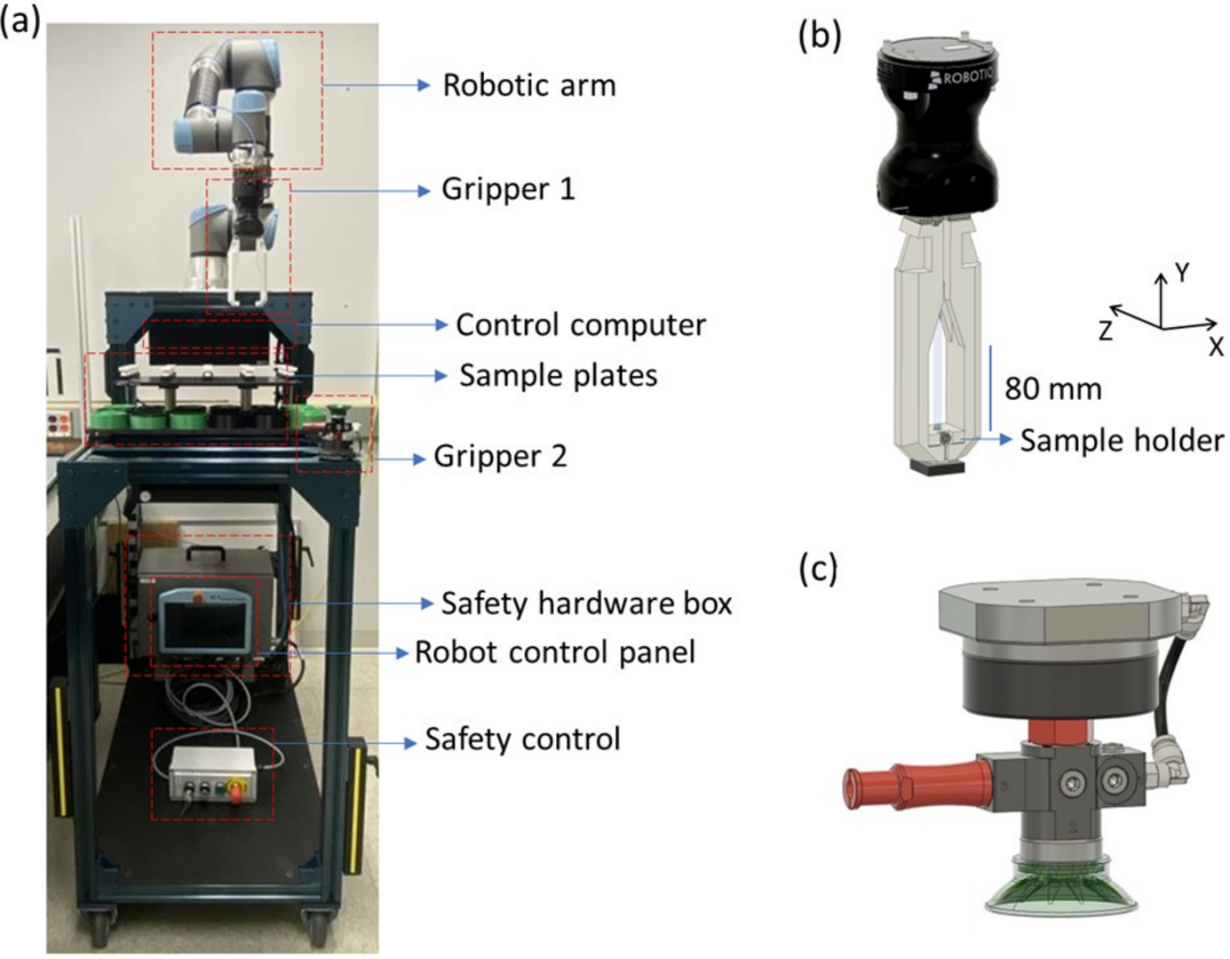
The design of the Tomography Object Mounter (TOM) robotic arm cart. (*a*) A photograph of the robotic arm cart with all its hardware. (*b*) The detailed structure of gripper 1 used for holding small samples on the kinematic mount. (*c*) The detailed structure of the vacuum-based suction gripper 2 used for holding large and heavy samples.

A Universal Robotics (UR5e) robotic arm is used in this work to enable automatic sample exchange. To ensure flexibility and adaptability across various beamlines, the robotic arm is mounted on a portable cart equipped with all necessary accessories [Fig. 1 ▸(*a*)]. The robotic arm is positioned on an elevated frame to allow the robot effectively to grasp tall samples from the top. Such a design minimizes the risk of operating the robot near singularities or at extreme joint angles and prevents collisions with the cart frame. The TOM system also holds the sample plates, which conserves space within the robot’s working envelope. Two types of sample plate are available: one for small samples on kinematic mounts, and another for large or heavy samples, such as field-collected soil cores. The design is compatible with most types of sample analyzed on X-ray micro-CT beamlines. The small sample plate currently accommodates up to 20 samples simultaneously, while the large sample plate can hold 18 samples.

Depending on the size or shape of a large sample, the sample holder can be 3D-printed and mounted on the plates. In this experiment specifically, we designed a 3D-printed cylindrical sample holder to match the soil core diameter.

To handle this variety of samples, we implemented two different robot grippers. As shown in Fig. 1 ▸(*b*), we designed a 3D-printed gripper and sample mount base on kinematic mounts (Thorlabs, Newton, USA). The gripper holds the mount base from the sides. It works for samples with sizes smaller than 20 mm (*X*) × 20 mm (*Z*) and a maximum height (*Y*) of 80 mm, which accommodates most types of sample measured on synchrotron X-ray micro-CT beamlines. For heavy and large samples, we employ a vacuum-based suction gripper [Fig. 1 ▸(*c*)]. The gripper secures the sample via a venturi device using a source of conventional compressed air, without the need for mechanical clamping that might introduce damaging mechanical stress to delicate samples. Since the sides of the sample are not gripped with this system, the suction gripper also allows a higher density of samples to be placed on the sample plate.

An important aspect of the TOM robotic cart design is the integration of safety controls and hardware to comply with beamline safety requirements. The safety interlocks include a beamline hutch door safety interlock which only allows normal robot operation while the door is closed. For development and alignment purposes, the safety controller allows for a keyed bypass of the door interlock along with keyed robot mode controls.

**Figure 2:**
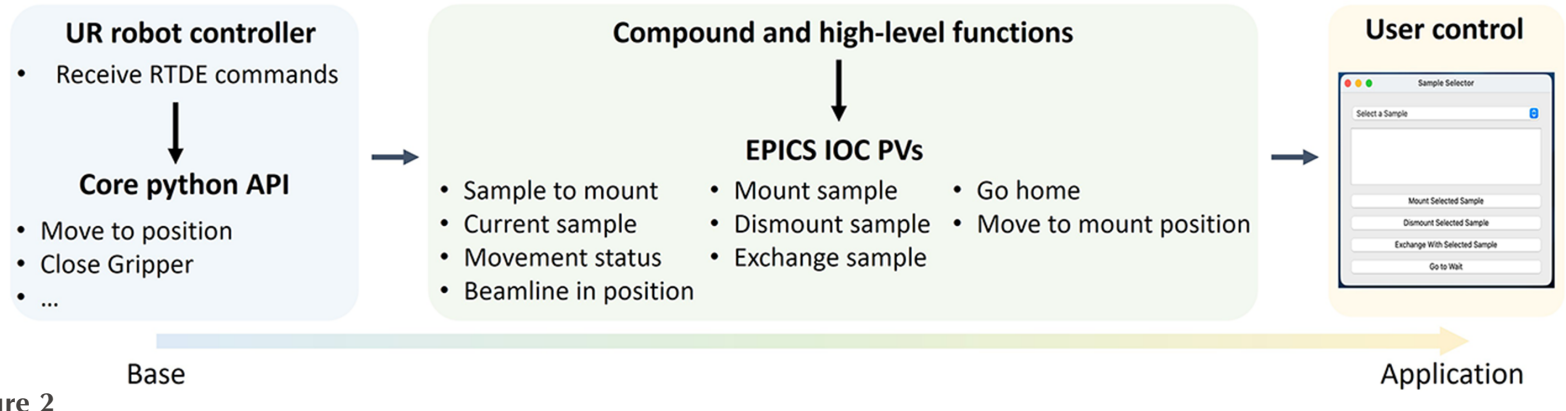
The layered software architecture of the robotic arm control.

For software control, the robot operation is managed by a layered software architecture. A schematic diagram of the robotic arm control from low-level hardware control to general user interface operation is shown in Fig. 2 ▸. At the lowest level, the robot controller executes real-time motion via real-time data exchange (RTDE) communication utilizing the *UR-RTDE* C++/Python package (Lindvig *et al.*, 2025 ▸). The core Python application programming interface (API) utilizes this package to provide I/O primitives and execution of accessible RTDE functions. This first layer uses this method to create well defined base functions, including get_position, move_to_position (linear move), open/close_gripper and other necessary core robotic functions. From these base functions, task-level and compound functions are composed and organized into semantically meaningful functional categories: (i) state and status handling, including sample to mount, sample identification and beamline readiness; (ii) sample handling, comprising (un)mount and exchange of sample; and (iii) positioning and homing, including homing routine and motion to the mount position of the robotic arm. The final level further expands protections, error handling and beamline-specific control, such as ensuring beamline hardware is in the proper location before robot movement. The layered software design has two advantages. First, it ensures that the RTDE layer and robot state feedback are isolated and only rely on the core python API, which is easy to maintain, and secondly, it helps to build up flexible and reproducible robot operations via compound functions. At the top level, the compound robot functions are exposed to the beamline control system through *Experimental Physics and Industrial Control System* (*EPICS*) process variables (PVs). Each PV is a compound function, and the execution states and fault reporting can be seen from the beamline control. These *EPICS* controls are easily integrated with the *EPICS* beamline controls. Thus, the robot actions are fully synchronized into the beamline, enabling automation coupling with other beamline components such as shutters and motor stages. To simplify interaction with the user, we further built a graphical user interface (GUI) to operate the robot.

### 2.3. Tomographic image resolution measurement

The resolution was quantified using a MicroCT Bar Pattern Phantom fabricated by QRM (Möhrendorf, Germany), which consists of two perpendicular silicon chips. Different types of patterns are etched on each silicon chip, including sets of specified line pairs and dots with sizes from 1 to 10 µm, a Siemens star, and a slanted edge with a depth range from 5 to 15 µm. A total of 2560 projections were collected over a 180° rotation with an exposure time of 0.01 s per frame. The corresponding results are discussed in Section 3.1.

### 2.4. High-throughput experiment on soil cores

**Figure 3:**
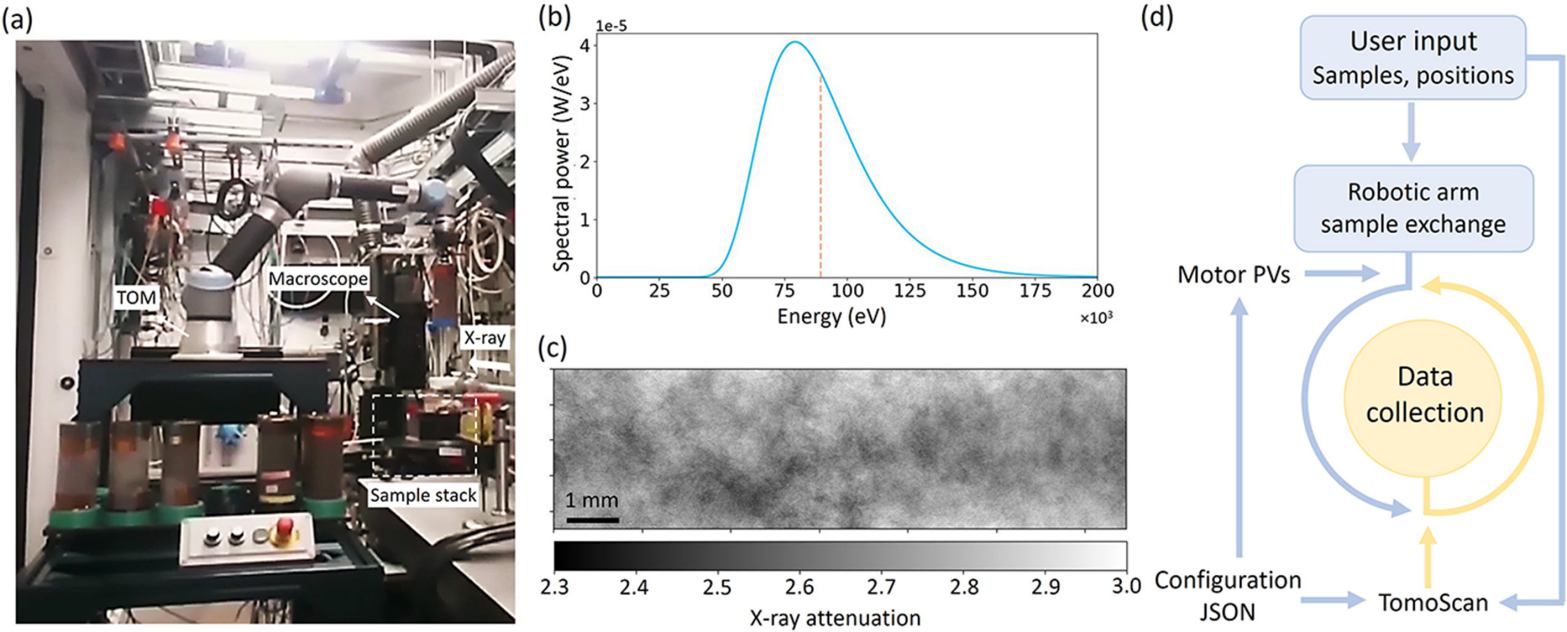
Demonstration of the high-throughput data collection on soil cores. (*a*) The experimental setup on the beamline with the integration of the TOM robotic arm cart. (*b*) The spectral power distribution as a function of photon energy with 3 mm Cu filters on 7-BM. (*c*) A representative flat-field-corrected projection of the sample showing X-ray attenuation. (*d*) The workflow for automatic sample exchange, movement and data collection.

The Molecular Observation Network (MONet) is an open science network developed by the Environmental Molecular Sciences Laboratory (EMSL) at Pacific Northwest National Laboratory (PNNL). It facilitates the collection of soil cores using a standardized sampling kit and field protocols (Bowman *et al.*, 2023 ▸), with two intact cores from each geographical location collected using a slide hammer soil corer (AMS, American Falls, USA). In this study, sixteen soil core samples with diameters of 76.2 mm and heights of 255 mm (Fig. S1 in the supporting information) from MONet have been measured in one automated process. The experimental setup and integration of the TOM robotic arm cart on 7-BM are shown in Fig. 3 ▸(*a*). The white beam was filtered with 3 mm of Cu, yielding a spectrum with a mean energy of 92.9 keV [Fig. 3 ▸(*b*)], as calculated in *OASYS* (Rebuffi & Sanchez del Rio, 2017 ▸). The TOM robotic arm cart is fixed next to the sample stack with the sample plate on the upstream side, so the robot movement path is clear with no risk of crashing into beamline components. The macroscope imaging system is placed ∼420 mm downstream from the sample, allowing for a significant amount of phase contrast. The imaging system was configured for 2× magnification, which resulted in an effective pixel size of 3.44 µm and a FOV of 11.1 mm (W) × 3.1 mm (H), with the vertical size limited by the natural width of the bending magnet (BM) beam in the vertical direction. For each scan, 2560 projections were collected over a 180° rotation with an exposure time of 0.06 s per frame, resulting in a scan time of less than 3 min. A representative flat-field-corrected projection [Fig. 3 ▸(*c*)] of the sample indicates the mean and maximum absorptions through the 76.2 mm soil core are 93.4% and 95.2%, respectively, corresponding to significant attenuation by the soil core. However, the reconstructed volumes from such fast data collection exhibit well preserved contrast and structural visibility, as discussed in the following section. To reconstruct the tomographic images, the GPU-based *TomocuPy* (Nikitin, 2023 ▸) reconstruction package was used with a Shepp–Logan filter and Paganin phase retrieval (Paganin *et al.*, 2002 ▸).

For the high-throughput experiment, the automation integrates sample exchange, sample positioning and data acquisition at three locations separated vertically by 75 mm, controlled via *EPICS* and *TomoScan* (Rivers & Carlo, 2023 ▸). Prior to the experiment, all samples are loaded on the TOM robotic arm cart sample plate. The robotic sample mounting position on the beamline is manually programmed when the cart is locked in place. Taking advantage of predefined relative geometry, the robot automatically computes the positions for the remaining sample variability without additional alignment. The beamline mounting configuration consists of coordinated motions to the sample table, hexapod *X* and *Y* axes, rotation stage, and sample stage *X* and *Z* axes. Thus, a safety interlock is implemented to verify the designated positions of all stages before robot motion is executed, mitigating the risk of collisions. After alignment, sample transport is initiated by selecting the corresponding position number on the sample plate.

The automation workflow is shown in Fig. 3 ▸(*d*). A worksheet with sample information and corresponding sample position number is prepared and loaded first. A separate PV configuration JSON file defines the PVs and target values for motor verification and motion control, which retains the flexibility to accommodate different experimental requirements. The scanning parameters are configured in *TomoScan*. At the start of the automated sequence, the first sample information from the worksheet is written to the *TomoScan* filename PV. Meanwhile, the corresponding sample position on the sample plate is input to the robot PV sample_to_mount, triggering the robot to transfer the sample to the beamline mounting position. The sample table is then moved to the first defined position and the *TomoScan* acquisition is initiated. Upon completion of the scan, the sample table is moved to the next position and the scan is repeated. Once the data collection at all three positions is completed, the sample stack returns to the mounting position to enable robotic exchange to the next sample (Video S1). The high-throughput process continues iteratively until all samples listed in the worksheet have been processed.

## 3. Results

### 3.1. Tomographic image spatial resolution

**Figure 4:**
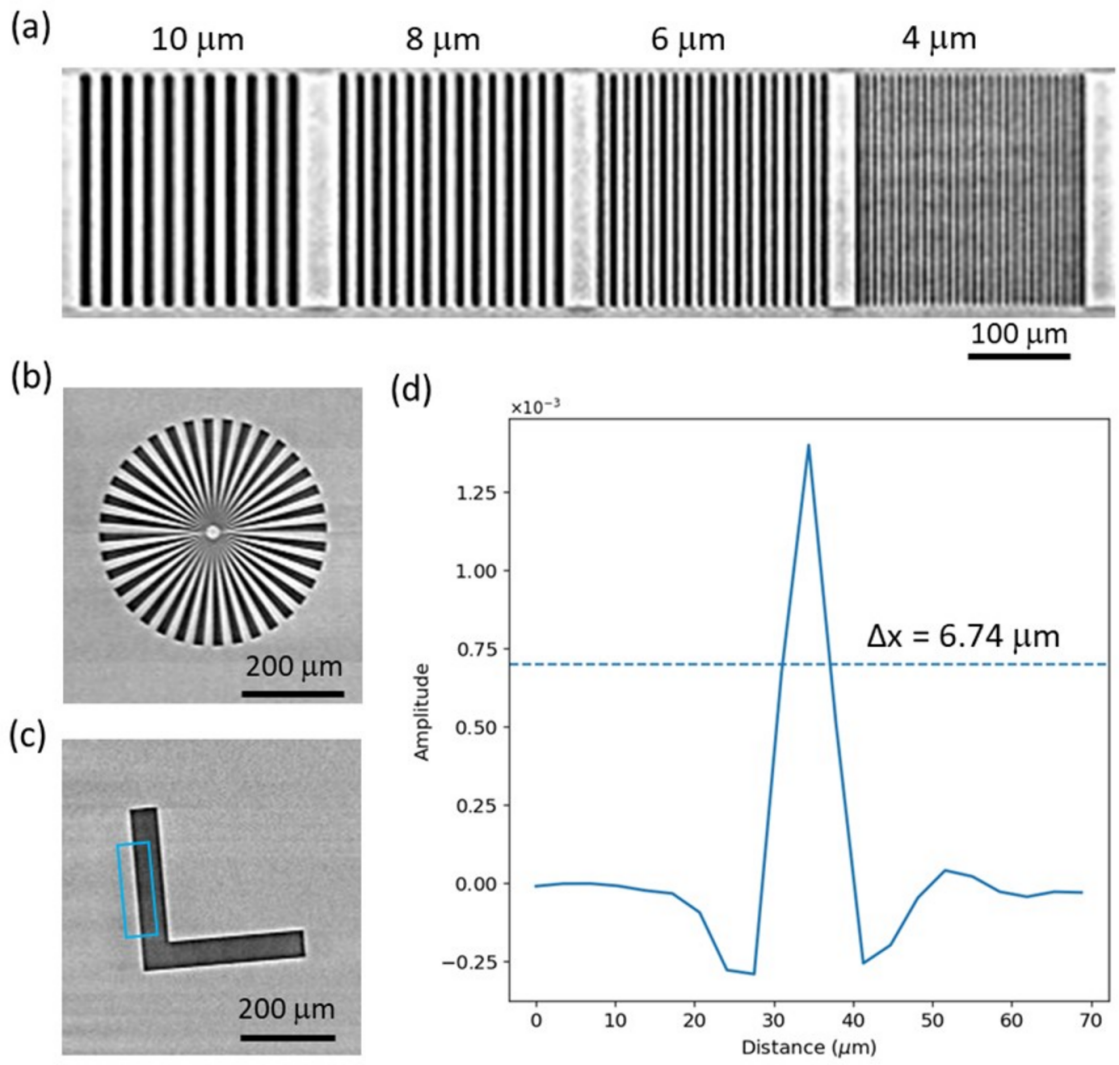
Verification of the resolution of the reconstructed image using the QRM bar pattern phantom. (*a*) Line pairs with widths ranging from 4 to 10 µm. (*b*) The reconstruction of the Siemens star. (*c*) The reconstruction of the slanted L edge. (*d*) The LSF plot calculated as the first derivative from the highlighted frame in blue in panel (*c*).

The resolution calculation was conducted on the reconstructed 3D tomographic images. Because the resolution measurement uses the same beam filtering, sample-to-detector distance and reconstruction parameters (Shepp–Logan filter and Paganin phase retrieval) as the soil samples, it reflects the resolution that can be obtained during normal acquisitions. Fig. 4 ▸(*a*) shows the line pairs with individual line widths ranging from 4 to 10 µm. The line pairs with widths of 6, 8 and 10 µm show distinct image contrast, while the line pairs with a width of 4 µm are slightly blurred, which suggests the imaging system can resolve ∼6 µm features. Similar resolution and image contrast can be seen on the Siemens star pattern [Fig. 4 ▸(*b*)]. Figs. 4 ▸(*c*) and 4 ▸(*d*) show the reconstructed slanted edge section and the corresponding line spread function (LSF) calculation from the edge response [Fig. S2(*a*)]. The full width at half-maximum (FWHM) of the LSF is 6.74 µm and the modulation transfer function (MTF) drops to 0.1 at 0.13 line-pairs µm^−1^ [Fig. S2(*b*)]. These results indicate that, under the current beamline and reconstruction configuration, the reconstructed image is capable of resolving features with dimensions of the order of two voxels. The resolution measurement here provides a direct and quantitative benchmark for the spatial resolution of the soil core experimental configuration. In the following section, representative images further demonstrate that the imaging system can resolve pore and structural features while also providing sufficient contrast to differentiate among distinct phases.

### 3.2. Tomographic image analysis of the soil cores

**Figure 5:**
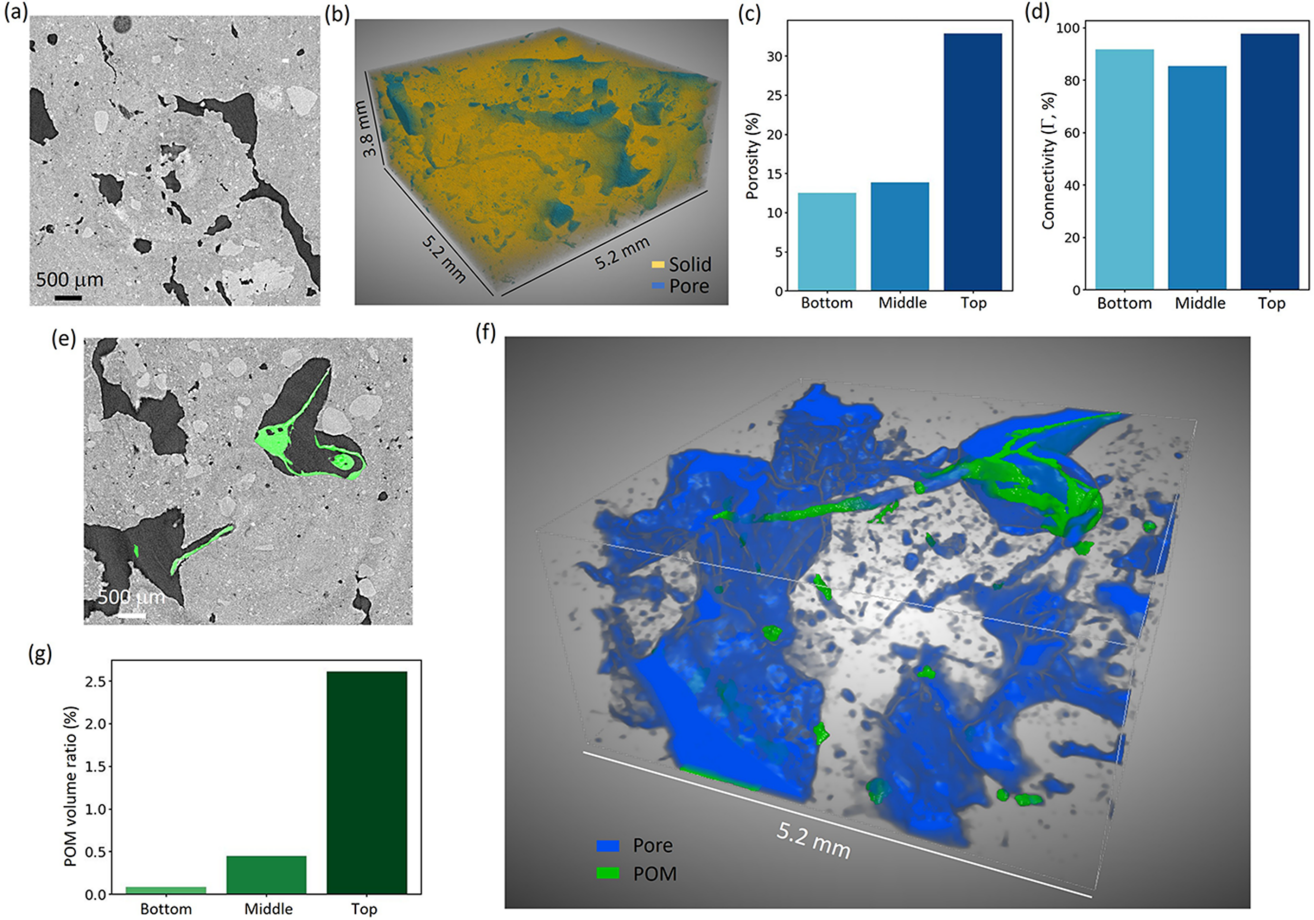
Image analysis and quantification of tomographic images of a representative soil core. (*a*) A 2D image of the scan in the middle section of the soil core. (*b*) A 3D visualization of the middle section. (*c*) Porosity comparison in the bottom, middle and top sections of the soil core. (*d*) A comparison of the connectivity (Γ) of the air-filled pores. (*e*) A representative segmentation of particular organic matter (POM) highlighted in green in a 2D image. (*f*) A visualization to show the interaction between air-filled pores and POM. (*g*) A comparison of the volume ratio of POM.

The tomographic image analysis was conducted on a representative soil core from Symsonia, Kentucky. Figs. 5 ▸(*a*) and 5 ▸(*b*) and Video S2 display a 2D slice and a 3D visualization, respectively, of the reconstructed soil volume. The images show strong contrast between air-filled pores and the mineral matrix, evident from the two distinct peaks in the histogram (Fig. S3) representing these phases. This image quality permits simple histogram-based thresholding to segment air-filled pores. Visible porosity and pore connectivity were calculated for the bottom, middle and top sections of the core [Figs. 5 ▸(*c*) and 5 ▸(*d*)]. Here, ‘visible’ refers to pore space resolved at the imaging resolution; pores smaller than two voxels are not detected and are therefore excluded. Visible porosity was 12.5%, 14.0% and 33.0% for the bottom, middle and top, respectively. This depth-dependent trend is driven by two factors. First, near-surface soil horizons contain more organic matter from plant roots and leaf litter which promotes biological activity from soil fauna, which creates visible macropores. Second, clay content increases with depth while sand content decreases. Although this textural shift tends to increase total porosity, it moves the pore size distribution towards sub-micrometre pores that fall below the imaging resolution, so the visible pore fraction decreases. Pore connectivity was not strongly driven by soil depth for this sample, with the pore connectivities being 91.8%, 85.3% and 97.7% for the bottom, middle and top, respectively. Despite lower visible porosity at depth, the pores are still highly connected due to continuous features such as cracks and pores of biological origin.

Particulate organic matter (POM) consists of plant roots and litter through various stages of decomposition and is of great interest to soil science, but is challenging to isolate in X-ray CT for two reasons: its attenuation coefficient lies between mineral and air phases, and voxels at pore–mineral interfaces fall in the same intermediate grayscale range due to the partial-volume effect. Simple thresholding cannot reliably distinguish true POM, but the propagation-based phase contrast enhances the phase differentiation between POM and the mineral matrix to enable AI-based segmentation. The resulting images were used to train an nnU-Net model (Isensee *et al.*, 2021 ▸) for segmentation of large and continuous POM fragments. Fig. 5 ▸(*f*) shows the 3D distribution of both air-filled pores and POM in the middle section. POM fragments occupy the air-filled pore space rather than being embedded in the mineral matrix. The POM volume fraction was quantified across all three sections [Fig. 5 ▸(*g*)], showing the widely documented effect of decreasing POM abundance with depth (Jobbágy & Jackson, 2000 ▸): POM abundance in the top layer was approximately ten times greater than in the bottom layer, reflecting reduced root and litter incorporation with depth.

The samples in this study are part of MONet, which aims to build a continental-scale, structured and searchable database of standardized high-value molecular and microstructural data from sediments, soils and critical mineral sources. Quantitative pore analysis derived from high-throughput synchrotron X-ray micro-CT will be integrated into the database, together with a broad range of complementary measurements including bulk density, gravimetric water content, pH, extractable phospho­rus, water- and HCl-extractable C and N, microbial biomass C and N, organic matter composition by Fourier transform ion cyclo­tron resonance mass spectrometry (FT-ICR-MS), microbial community composition via metagenomic sequencing, soil texture, respiration, extractable inorganic N, exchangeable base cations and cation exchange capacity, bioavailable metals, extractable sulfate and aluminium, and hydraulic properties. Because these data are generated using standardized protocols and instrument platforms, they are harmonized across sites and released as open, FAIR (findable, accessible, interoperable, reusable) (Wilkinson *et al.*, 2016 ▸) datasets through EMSL-hosted and Department of Energy supported data portals for use in machine learning and modeling of microbially mediated biogeochemical processes from pore to continental scales.

## 4. Conclusion and future work

In the present work, we have demonstrated an automated synchrotron X-ray micro-CT workflow including sample exchange, movement and data collection on the 7-BM beamline at the APS. A mobile robotic arm cart has been developed that integrates hardware and software control into a single transportable platform. It can be deployed and operated rapidly across multiple beamlines for different types of sample, enabling automatic sample exchange. Together with recent beamline developments, which include a high NA imaging system and large motor travel, we have achieved high-throughput data collection from soil cores with minimal physical human intervention. The implementations can be easily adapted to different types of sample and routinely operated on the beamline for users.

Future work can extend the automation to image reconstruction. There are some available algorithms (Vo *et al.*, 2014 ▸; Vo *et al.*, 2021 ▸; Vacek & Jacobsen, 2022 ▸) for determining the CoR for tomographic reconstruction, which can be evaluated to identify the CoR automatically and perform image reconstruction. When coupled with the APS data management system (Veseli *et al.*, 2018 ▸), the reconstructed data can be automatically uploaded to APS data storage and made available to users in real time. The current automation applies to samples larger than the FOV; samples smaller than the FOV require additional sample alignment. Further integration of computer vision or AI-based recognition techniques will address these limitations, thereby extending the application of this high-throughput micro-CT workflow to a broader range of samples.

## Supplementary Material

Video S1: recording of the automated experiment on soil cores at 7BM, APS. DOI: 10.1107/S1600577526005539/tol5024sup1.avi

Video S2: 3D visualization of the middle section of the representative soil core. DOI: 10.1107/S1600577526005539/tol5024sup2.avi

Supporting Figures S1 to S3. DOI: 10.1107/S1600577526005539/tol5024sup3.pdf
