# Supplementary material for: A robotic and high-throughput X-ray micro-computed tomography workflow
Source: J Synchrotron Radiat. 2026 Jun 22;33(Pt 4):1190–7. doi: 10.1107/S1600577526005539 (PMC13344543; doi:10.1107/S1600577526005539)
Supplement: Supplementary file 3 [file s-33-01190-sup3.pdf]

JOURNAL OF SYNCHROTRON RADIATION

Volume 33 (2026)

Supporting information for article:

A robotic and high-throughput X-ray micro-computed tomography workflow

Xiaoyang Liu, Alex Lavens, James Bennett O'Sullivan, Alan Kastengren, Andrew T. Townsend, Jason G. Toyoda, Hasitha Wijesuriya, Tamas Varga and Karolina Michalska

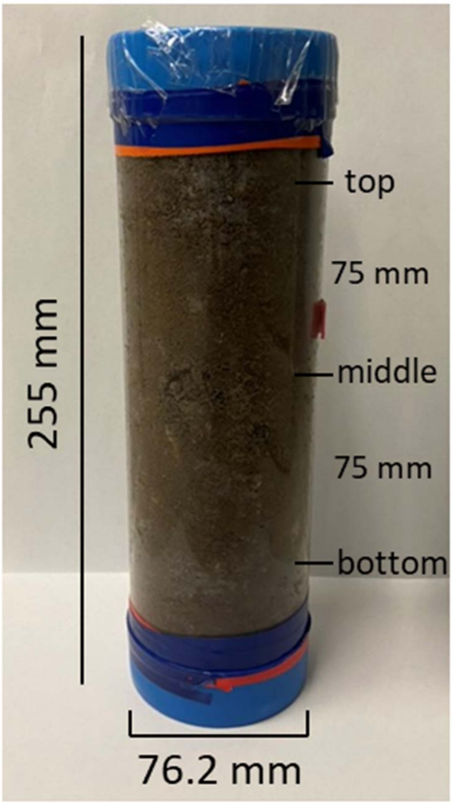

Figure S1 The soil core used in the work with defined scan positions.

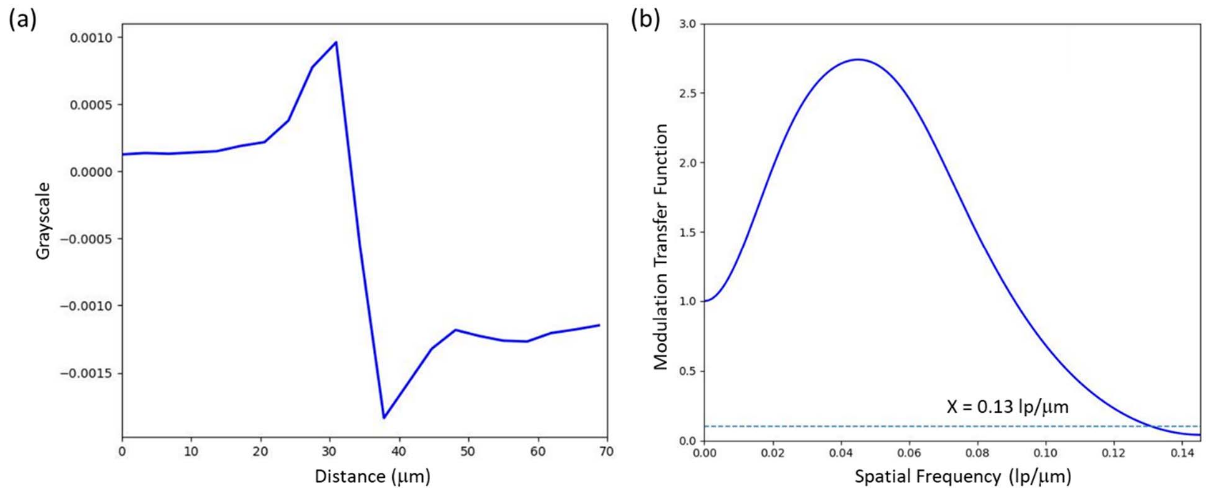

Figure S2 The slanted edge response from the highlighted frame of reconstructed image in Figure 3 c. (a) The grayscale value change across the slanted edge. (b) The modulation transfer function (MTF) calculated from LSF from the edge. The 0.1 MTF amplitude is at 0.13 lp/μm.

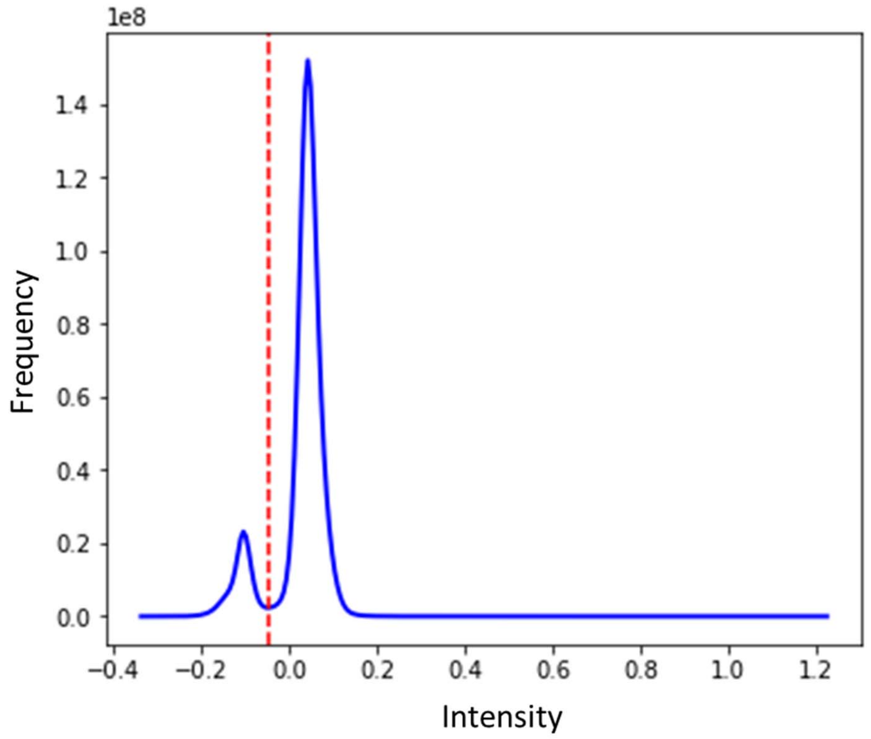

Figure S3 Histogram of the tomographic image data shown in Fig. 5.
